# Development of a Foliar Synergist Based on Radiation-Synthesized Potassium Polyacrylate for Rice Yield Enhancement

**DOI:** 10.3390/polym18141721

**Published:** 2026-07-13

**Authors:** Lu Liu, Hongrui Wang, Caifeng Zhao, Weiliang Zhang, Hongke Xie, Jianliang Tang, Leping Zhang, Yuan Yuan, Longxin Jin, Sai Shao

**Affiliations:** 1 Hunan Institute of Nuclear Agriculture Sciences and Chinese Herbal Medicines, Changsha 410125, China; lliu1994@hunaas.cn (L.L.); hrwang@hunaas.cn (H.W.); cfzhao1988@163.com (C.Z.); xhksee@hunaas.cn (H.X.);; 2 Hunan Ava Seed Industry Co., Ltd., Changsha 410125, China; zhangweiliang119@126.com (W.Z.); tangjianliang@lpht.com.cn (J.T.); yuanyuan@lpht.com.cn (Y.Y.)

**Keywords:** potassium polyacrylate, foliar application, rice yield, moisture retention, nutrient delivery

## Abstract

Stable rice production is critical for ensuring national food security and agricultural sustainability. Climate change is increasing the demand for efficient crop management strategies to maintain rice production. Foliar fertilization enables rapid nutrient supplementation by directly delivering nutrients to aboveground tissues while avoiding soil-related limitations. However, most foliar formulations are primarily designed for rapid nutrient delivery and have limited capacity to prolong water retention and nutrient availability on leaf surfaces after application. Hydrogels possess excellent water-retention and nutrient-delivery capabilities, but their intrinsic crosslinked networks limit water solubility and foliar suitability. Inspired by these characteristics, a sprayable polymer-based formulation was designed to combine hydrogel-like moisture preservation with foliar application compatibility. In this study, a foliar moisture-preserving synergist (FMPS) was developed using radiation-synthesized potassium polyacrylate as the polymer matrix, with urea and glucose incorporated as nitrogen and carbon sources, respectively. Structural characterization revealed morphological changes after incorporation of urea and glucose into PAA-K, while Fourier transform infrared spectroscopy suggested their incorporation and possible intermolecular interactions. Under standard growth conditions, FMPS increased the effective panicle number, filled grain number, and seed-setting rate by 29.3%, 18.4%, and 4.0%, respectively, resulting in significantly improved rice yield. These findings demonstrate the potential of FMPS as a hydrogel-inspired foliar formulation for enhancing rice productivity.

## 1. Introduction

The stable production of rice, which is one of the most widely consumed grains globally, is crucial to national food security and sustainable agricultural development [1]. However, climate change is increasing environmental variability, posing growing challenges to rice production systems [2,3,4]. The Yangtze Basin, one of the most important rice-producing regions in China, is characterized by fertile plains and abundant water resources, yet rice production in this region is increasingly affected by periodic heat and water stress during the growing season [5,6]. Previous studies have estimated that a 1 °C increase in temperature may reduce rice yield by approximately 10% [7]. This vulnerability was highlighted during 2022, when a heatwave and drought event in the Yangtze River Basin affected 52.45 million people and caused direct economic losses exceeding 51 billion yuan [8]. These challenges underscore the importance of developing efficient agronomic practices to sustain rice productivity under changing environmental conditions.

Foliar fertilization has emerged as an effective strategy for improving nutrient use efficiency and maintaining crop productivity. By directly delivering nutrients to aboveground tissues, it bypasses soil-related constraints such as nutrient fixation and limited root uptake, allowing rapid supplementation during critical growth stages [9,10,11,12]. In particular, foliar application of carbon and nitrogen sources has also been reported to promote plant growth and physiological responses [13,14]. However, most foliar formulations are designed primarily for rapid nutrient delivery and have limited capacity to prolong water retention and nutrient delivery on leaf surfaces after application.

Recent studies have shown that hydrogels, owing to their three-dimensional networks and hydrophilic functional groups, are ideal carriers for water and nutrient delivery [15,16,17,18,19]. Accordingly, several hydrogel-based systems have been developed to provide sustained nutrient release and improve crop performance [20,21]. However, these hydrogel-based fertilizers are primarily designed for soil application [22,23], and their potential use as foliar spray formulations remains largely unexplored. Their intrinsic crosslinked network renders them insoluble in water, thereby preventing their direct application as sprayable formulations.

**Figure 1:**
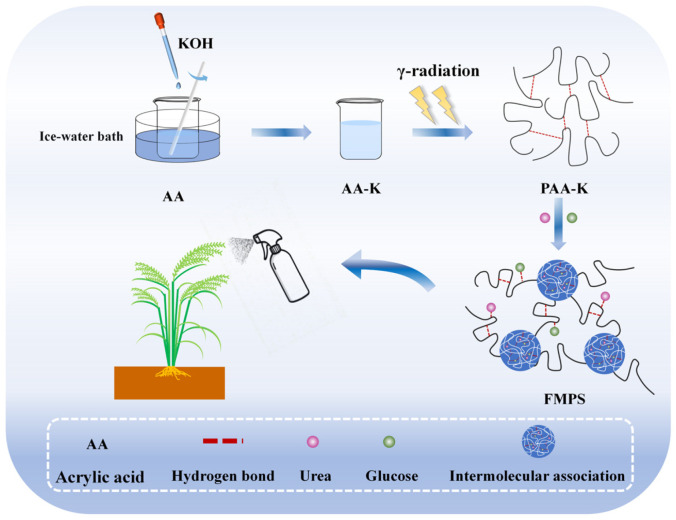
Schematic illustration of the preparation and application of FMPS.

To address this limitation, we proposed a design strategy based on a water-soluble linear polymer that remains fluid during spraying and is expected to form a gel-like network on leaf surfaces after drying, thereby enhancing moisture retention. To further integrate nutrient delivery with moisture preservation, urea and glucose were incorporated as nitrogen and carbon sources, respectively. Based on this design strategy, a foliar moisture-preserving synergist (FMPS) was developed using radiation-synthesized potassium polyacrylate (PAA-K) as the polymer matrix (Figure 1). The resulting formulation enables simultaneous foliar moisture retention and nutrient delivery, offering a promising strategy for enhancing rice yield.

## 2. Materials and Methods

### 2.1. Chemicals

Acrylic acid (AA, AR, >99%) was purchased from Macklin Biochemical Co., Ltd. (Shanghai, China). Potassium hydroxide (KOH, AR, ≥85%) was purchased from Tianjin Hengxing Chemical Preparation Co., Ltd. (Tianjin, China). Urea (AR, ≥99.0%), glucose (AR, ≥99.0%), and sodium dodecyl sulfate (SDS, CP) were purchased from Sinopharm Chemical Reagent Co., Ltd. (Shanghai, China). Deionized water was produced using an Eco-S15 laboratory water purification system (Shanghai Hetai Instrument Co., Ltd., Shanghai, China). All chemicals were used as received without further purification.

### 2.2. Preparation of PAA-K

Acrylic acid (AA) was placed in an ice-water bath, and a 30 wt% KOH aqueous solution was added dropwise under continuous stirring to achieve a neutralization degree of 80%. Sodium hypophosphite (0.2 wt%, based on the mass of AA) was subsequently added as a chain transfer agent to suppress excessive covalent crosslinking during irradiation, thereby favoring the formation of water-soluble linear PAA-K chains. After complete dissolution of the additives, the reaction mixture was sealed and irradiated using a ^60^Co γ-ray source (Hunan Institute of Nuclear Agriculture and Chinese Herbal Medicines, Changsha, China) at an absorbed dose of 5 kGy to initiate radiation-induced polymerization, yielding water-soluble potassium polyacrylate (PAA-K).

### 2.3. Preparation of FMPS

FMPS was prepared by sequentially adding PAA-K (43.2 wt%), urea (1.1 wt%), glucose (8.1 wt%), and a 25 wt% SDS solution (8.1 wt%) into deionized water (39.5 wt%) in a 250 mL beaker, resulting in a total formulation mass of 185.0 g. Urea was incorporated as a nitrogen source, glucose as a carbon source, and SDS as a surfactant to improve the wettability of the foliar spray. These components were incorporated according to their intended functions in the formulation and were expected to contribute to its overall performance. The mixture was stirred magnetically at 500 rpm for 10–15 min at 25 ± 1 °C until a homogeneous, transparent solution free of visible particles was obtained. The pH of the resulting formulation was measured at 25 ± 1 °C using a calibrated pH meter (Mettler Toledo S220, Greifensee, Switzerland) and was found to be 6.48.

### 2.4. Structural Characterization of PAA-K and FMPS

The microstructures of dried PAA-K and FMPS samples were characterized using a scanning electron microscope (SEM, MIRA CMS, Tescan, Brno, Czech Republic). Prior to imaging, samples were mounted onto conductive carbon tape and sputter-coated with gold for 45 s at 10 mA. SEM images were acquired at an accelerating voltage of 3 kV.

The elemental compositions were analyzed by energy-dispersive spectroscopy (EDS) using the same instrument at an accelerating voltage of 15 kV. The map sum spectrum was collected over an energy range of 0–20.48 keV with a channel resolution of 0.01 keV per channel and a live acquisition time of 43.3 s. Elemental distribution maps of C, O, K, and N were recorded.

Dynamic light scattering (DLS) measurements were performed using a Zetasizer Pro (Malvern Panalytical Ltd., Malvern, UK) equipped with a 633 nm He-Ne laser. PAA-K and FMPS samples were diluted with deionized water to the appropriate concentration. Each sample (1 mL) was transferred into a disposable cuvette and equilibrated at 25 °C for 2 min before measurement.

Fourier transform infrared spectroscopy (FTIR) was carried out using a Nicolet iS5 spectrometer (Thermo Fisher Scientific, Madison, WI, USA). Dried PAA-K powder was analyzed using the KBr pellet method (sample-to-KBr mass ratio of 1:100), whereas FMPS, as a liquid formulation, was analyzed using an attenuated total reflectance (ATR) accessory. Spectra were collected over the range of 4000–400 cm^−1^ at a resolution of 4 cm^−1^, with 32 scans.

The average molecular weight of PAA-K and the apparent molecular weight of FMPS were determined by gel permeation chromatography (GPC, PL-GPC50, Agilent Technologies, Santa Clara, CA, USA). Samples were prepared as 2 mg·mL^−1^ aqueous solutions and filtered through 0.45 μm syringe filters before analysis. Separation was performed on a PLgel Aquagel-OH Mixed-M column maintained at 35 °C using 0.1 mg·mL^−1^ NaNO_3_ aqueous solution as the mobile phase at a flow rate of 1.0 mL·min^−1^. The injection volume was 100 μL, and the total analysis time was 30 min. The eluted samples were monitored using a differential refractive index detector coupled with a laser light scattering detector.

### 2.5. Performance and Application Evaluation of FMPS

#### 2.5.1. Surface Tension Measurement

The surface tension of FMPS solutions was measured at 25 ± 1 °C using the Du Noüy ring method with a surface tensiometer (MC-1021, MinCe Instrument Equipment Co., Ltd., Xiamen, China). The stock solution was diluted 5-, 10-, 25-, 50-, 100-, and 150-fold with deionized water. Each diluted sample was equilibrated for 2 min before measurement. Surface tension was measured in triplicate, and the average value was reported. Before each measurement, the platinum ring was rinsed thoroughly with deionized water and flame-dried.

#### 2.5.2. Visual Evaluation of Dispersion Behavior

To evaluate the dispersion stability of FMPS upon dilution, the stock solution was diluted 5-, 10-, 25-, 50-, and 100-fold with deionized water. Aliquots of each diluted solution were transferred into transparent glass vials and visually examined for clarity and dispersion behavior. Representative photographs were captured under identical lighting conditions using a digital camera.

#### 2.5.3. Detached-Leaf Water Loss Assay

The moisture-retention capability of FMPS on rice leaves was evaluated using a detached-leaf water loss assay. Healthy rice leaves at the tillering stage were excised and cut into 5 cm segments. Each segment was uniformly sprayed to saturation with either a 100-fold diluted FMPS solution or deionized water (CK). Immediately after spraying, the initial fresh weight (W_0_) was recorded. The leaf segments were then placed on dry filter paper in Petri dishes and incubated under controlled laboratory conditions (25 ± 1 °C and 60 ± 5% RH). Fresh weights (W_t_) were recorded at 30, 60, 90, 120, 150, 180, 240, 300, 720, and 1440 min. Three independent biological replicates (*n* = 3) were included for each treatment. The water loss rate was calculated using the following equation:

$$Water\;loss\;rate\;(\%)=\frac{\left(W_{0}-W_{t}\right)}{W_{0}}\times 100$$

#### 2.5.4. Indoor Pot Experiment

Rice seeds of the cultivar ‘Chuangyu 9’ were germinated until radicle emergence and then sown in seedling trays (length × width × height: 440 mm × 290 mm × 80 mm) filled with paddy soil collected from the experimental field of the Hunan Academy of Agricultural Sciences. The soil had the following properties: pH 6.34, organic matter 37.7 g·kg^−1^, total nitrogen 2.1 g·kg^−1^, total phosphorus 1.2 g·kg^−1^, total potassium 12.0 g·kg^−1^.

At the three-leaf one-heart stage, seedlings were thinned. Thirty days later, uniform seedlings were transplanted individually into plastic pots (diameter × height: 280 mm × 190 mm), each containing 10 kg of paddy soil. Two treatments were established: water (CK) and FMPS. Each treatment consisted of 32 pots arranged in a completely randomized design. All pots received identical irrigation and fertilization management throughout the experiment. Based on the optimization results of the surface tension and dispersion evaluations, FMPS was diluted 100-fold with deionized water before foliar application. The first foliar spray was applied at the fourth stage of panicle differentiation, followed by a second application 7 days later.

After maturity, fifteen plants were randomly selected from each treatment and divided into three biological replicates (five plants per replicate). The effective panicle number, filled grain number, seed-setting rate, panicle length, 1000-grain weight, and grain yield of each biological replicate were determined.

### 2.6. Statistical Analysis

All experiments were performed with at least three independent biological replicates. Data are presented as the mean ± standard deviation (SD). Data processing and figure preparation were carried out using Microsoft Excel 2016 and Origin 2024. Statistical analyses were performed using IBM SPSS Statistics 26.0. Differences between the two treatments were evaluated using an independent-samples Student’s *t*-test, and *p* < 0.05 was considered statistically significant.

## 3. Results and Discussion

### 3.1. Preparation and Characterization of FMPS

**Figure 2:**
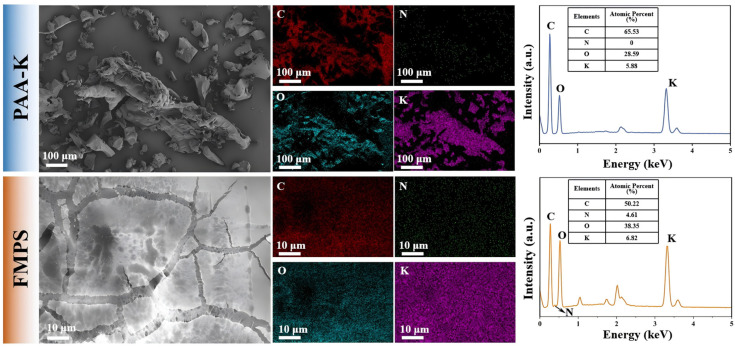
SEM images, EDS spectra, and corresponding elemental maps of PAA-K and FMPS.

FMPS was prepared by incorporating urea and glucose into radiation-synthesized potassium polyacrylate (PAA-K). The morphology and physicochemical properties of PAA-K and FMPS were subsequently characterized using SEM, EDS, DLS, FTIR, and GPC. SEM images and corresponding EDS elemental mappings (Figure 2) revealed distinct differences between PAA-K and FMPS. SEM images showed that the freeze-dried PAA-K exhibited a three-dimensional network, which may contribute water retention through hydrophilic interactions [24]. After the incorporation of urea and glucose, FMPS exhibited a markedly smoother and more compact morphology than PAA-K. This morphological change suggests that the incorporated components were distributed within the PAA-K matrix during drying, partially filling the original structure and resulting in a denser architecture.

EDS elemental mapping further supported the successful incorporation of urea into FMPS. Compared with PAA-K, FMPS exhibited a distinct nitrogen (N) signal, while quantitative analysis showed that the atomic percentage of N increased from 0% in PAA-K to 4.61% in FMPS. Collectively, the SEM and EDS results support the successful incorporation of urea into the FMPS formulation.

**Figure 3:**
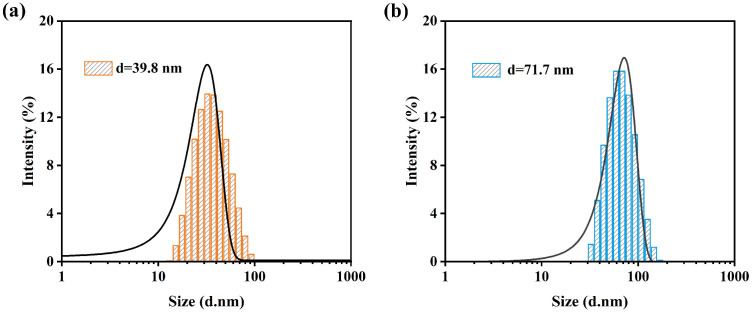
DLS size distribution of PAA-K (**a**) and FMPS (**b**). The black solid line represents the fitted particle size distribution curve.

DLS analysis was employed to characterize the hydrodynamic diameterof PAA-K and FMPS in aqueous solution (Figure 3). The average hydrodynamic diameter of PAA-K was 39.8 ± 14.1 nm (PDI = 0.14), whereas FMPS exhibited an increased diameter of 71.7 ± 27.7 nm (PDI = 0.17). This approximately 1.8-fold increase in hydrodynamic diameter suggests that the incorporation of urea and glucose altered the solution-state organization of the formulation. One possible explanation is the formation of intermolecular associations between PAA-K and the incorporated small molecules. Specifically, the carboxylate groups of PAA-K, together with the amino and carbonyl groups of urea and the hydroxyl groups of glucose, may participate in hydrogen-bonding interactions, which may contribute to the formation of larger solution-state assemblies. However, these interactions were not directly investigated in the present study. Both samples exhibited low PDI values (<0.20), indicating relatively narrow size distributions and good dispersion uniformity. Overall, the DLS results suggest that the incorporation of urea and glucose increased the hydrodynamic size while maintaining good dispersion characteristics in aqueous solution.

**Figure 4:**
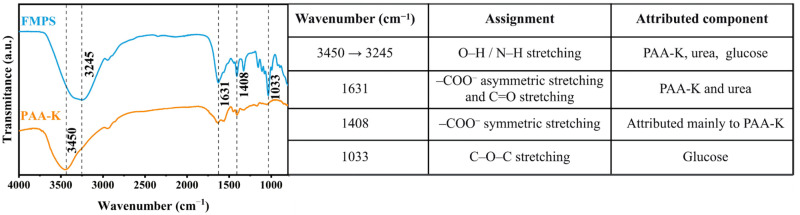
FTIR spectra of PAA-K and FMPS.

To further examine the possible interactions among FMPS components, FTIR analysis was performed (Figure 4). Compared with PAA-K, the FTIR spectrum of FMPS exhibited several distinct changes. The broad absorption band near 3450 cm^−1^ shifted to a lower wavenumber (3245 cm^−1^), suggesting changes in the O−H and N−H stretching vibrations. This shift may be associated with hydrogen-bonding interactions involving the amino groups of urea, the hydroxyl groups of glucose, and the carboxyl and carboxylate groups of PAA-K [25]. Compared with PAA-K, the FMPS spectrum exhibited a more pronounced absorption peak at 1631 cm^−1^, which may arise from the overlapping contributions of the asymmetric stretching vibration of the carboxylate groups in PAA-K [26] and the C=O stretching vibration of urea [27]. In addition, the absorption band at 1408 cm^−1^ became noticeably more intense after the incorporation of urea and glucose, although the individual contributions of different functional groups cannot be distinguished based on the present FTIR data alone. A stronger absorption peak was also observed at 1033 cm^−1^, corresponding to the C−O stretching vibration of glucose [28], which is consistent with the incorporation of glucose into the FMPS formulation. Collectively, these spectral changes support the successful incorporation of urea and glucose into FMPS and are consistent with the possible presence of intermolecular hydrogen-bonding interactions.

**Figure 5:**
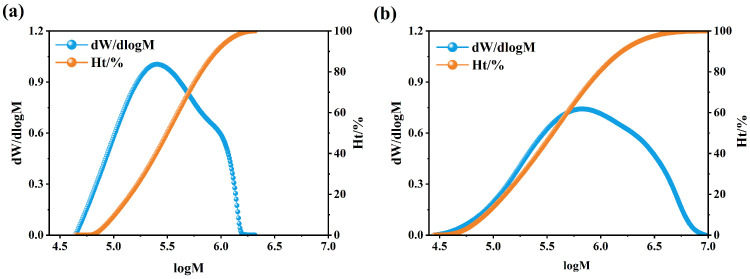
GPC traces of (**a**) PAA-K and (**b**) FMPS. log M represents the logarithm of molecular weight, dW/dlogM denotes the weight fraction per logarithmic molecular weight interval, and HT% represents the percentage of cumulative height.

GPC analysis was further carried out to evaluate the apparent molecular characteristics of FMPS in aqueous solution (Figure 5). Sodium hypophosphite (0.2 wt%) was employed as a chain transfer agent, while γ-irradiation polymerization produced water-soluble PAA-K with an apparent molecular weight of approximately 4.0 × 10^5^ g·mol^−1^. After the incorporation of urea and glucose, the apparent molecular weight increased to approximately 1.2 × 10^6^ g·mol^−1^. This increase suggests that the incorporation of the functional components altered the solution-state organization of the formulation. One possible explanation is the formation of intermolecular associations involving PAA-K, urea, and glucose, which increase the apparent hydrodynamic volume and result in higher apparent molecular weight values duringGPC analysis [29,30]. Meanwhile, the PDI increased from 1.84 to 3.10, indicating a broader apparent molecular weight distribution following the incorporation of the functional components. Together with the DLS and FTIR results, these observations are consistent with the possibility that intermolecular hydrogen bonding occurs among the formulation components in aqueous solution.

Collectively, these results support the successful incorporation of urea and glucose into the FMPS system. The coordinated changes in morphology, solution-state properties, and FTIR characteristics suggest the occurrence of intermolecular interactions, with hydrogen bonding potentially contributing to the structural organization and physicochemical properties of FMPS. However, the detailed molecular interaction mechanism remains to be elucidated.

### 3.2. Application Performance of FMPS

**Figure 6:**
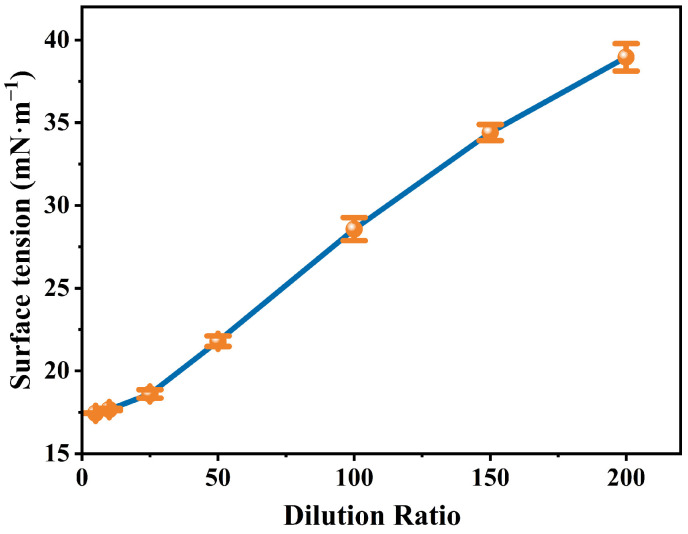
Surface tension of FMPS solutions at different dilution ratios.

Surface tension is a key factor governing the wetting and retention behavior of spray droplets on rice leaf surfaces [31,32]. To determine an appropriate application concentration, the surface tension of FMPS solutions at different dilution ratios was measured. As shown in Figure 6, the surface tension gradually increased with increasing dilution ratio. At a dilution ratio of 100-fold or lower, the surface tension remained below the reported critical surface tension of rice leaves (approximately 30 mN·m^−1^) [33], which favors droplet spreading on the leaf surface. In contrast, at dilution ratios greater than 100-fold, the surface tension increased to 34.41 mN·m^−1^, exceeding the critical value for rice leaves. Under these conditions, droplets are more likely to remain as discrete beads, reducing the contact area with the leaf surface and increasing droplet bouncing or runoff, thereby decreasing retention efficiency [34]. Therefore, dilution ratios of 100-fold or lower were considered suitable for subsequent foliar application.

**Figure 7:**
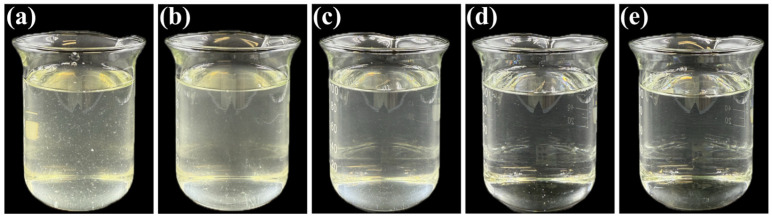
Photographs of FMPS solutions showing the dispersion behavior at different dilution ratios: (**a**) 5×, (**b**) 10×, (**c**) 25×, (**d**) 50×, and (**e**) 100×.

To further optimize the application concentration, the dispersion characteristics of FMPS at different dilution ratios were visually examined (Figure 7a–e). The appearance of the formulation changed significantly with an increasing dilution ratio. At dilution ratios below 100-fold, the formulation appeared less uniform dispersion, suggesting incomplete dispersion. In contrast, at dilution ratios of 100-fold or higher, the formulation formed a homogeneous, transparent, and visually stable dispersion. Considering both the surface tension and dispersion characteristics, a 100-fold dilution was selected for all subsequent foliar application experiments. At this dilution, FMPS exhibited favorable dispersion uniformity, which is expected to facilitate uniform spray deposition and leaf surface coverage. Accordingly, the 100-fold dilution was used in both the detached-leaf water loss assay and the pot experiment.

**Figure 8:**
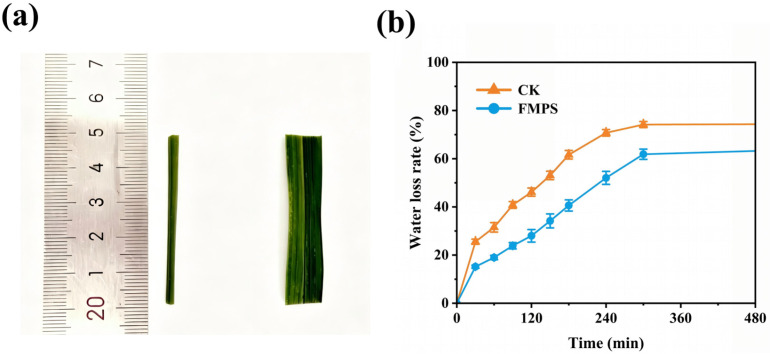
(**a**) Representative photographs of detached rice leaves sprayed with water, CK (**left**) and FMPS (**right**) after 1 h under ambient conditions. (**b**) Water loss rates of detached rice leaves treated with FMPS or deionized water (CK).

The moisture-retention performance of FMPS was evaluated using a detached-leaf water loss assay. As shown in Figure 8a, after 1 h of dehydration, the CK-treated leaf (left) exhibited obvious wilting and curling, whereas the FMPS-treated leaf (right) remained visibly more turgid and retained its structural integrity, indicating improved moisture retention on the leaf surface. The quantitative watermloss curves shown in Figure 8b further supported this observation. During the first 30 min, both treatments exhibited rapid weight loss, which was mainly attributed to the evaporation of residual spray solution on the leaf surface. By 60 min, the water loss rate of the FMPS-treated leaves was 19.0%, compared with 31.5% for the CK group. At 240 min, corresponding values were 49.4% and 70.8%, respectively, indicating that the moisture-retaining effect of FMPS persisted throughout the measurement period. Both curves gradually approached equilibrium after approximately 300 min, and the final water loss rates were 61.9% for FMPS and 74.2% for CK, corresponding to a persistent difference of 12.3 percentage points. These results demonstrate that FMPS effectively reduced water loss from detached rice leaves and maintained higher leaf moisture levels than the control throughout the measurement period, confirming the superior moisture-retention performance of FMPS following foliar application. The enhanced water retention may be related to intermolecular interactions among PAA-K, urea, and glucose, which may facilitate the formation of a gel-like layer on leaf surfaces upon water evaporation after foliar application.

### 3.3. Biological Effect Evaluation of FMPS

**Figure 9:**
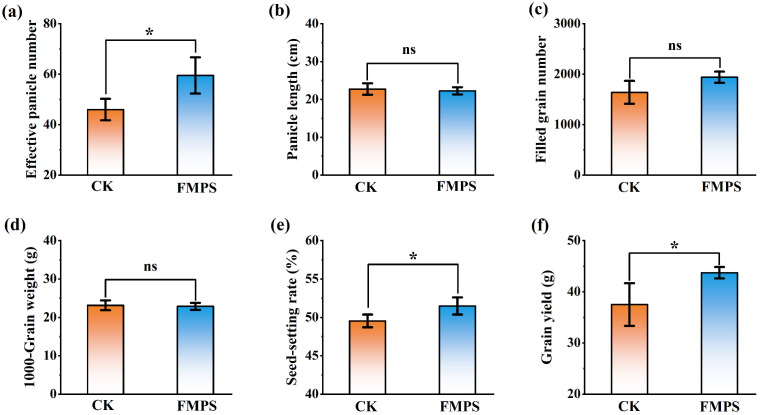
Effects of FMPS on rice yield-related traits under pot experiment conditions. (**a**) Effective panicle number; (**b**) Panicle length; (**c**) Filled grain number; (**d**) 1000-grain weight; (**e**) Seed-setting rate; (**f**) Grain yield. Data are presented as the mean ± SD (*n* = 3). * indicates a significant difference (*p* < 0.05), and ns indicates no significant difference (*p* ≥ 0.05).

A pot experiment was conducted to evaluate the effect of FMPS on rice yield under standard growth conditions. As shown in Figure 9, FMPS significantly enhanced several yield-related traits compared with the CK treatment. The average effective panicle number increased from 46.0 to 59.5, corresponding to a 29.3% increase. The average filled grain number increased from 1639 to 1940 grains, corresponding to an 18.4% increase compared with CK., while the seed-setting rate increased by 4.0 percentage points to 51.5%. No significant differences were observed in panicle length or 1000-grain weight, which is consistent with the findings of Sekiya et al. [35], who reported that increasing nitrogen supply does not necessarily affect 1000-grain weight. Consequently, the grain yield increased significantly by 16.5% compared with the CK treatment.

Therefore, we propose the following hypothesis to explain the observed yield improvement. Following foliar application, evaporation of water from the deposited droplets may facilitate intermolecular associations among PAA-K, urea, and glucose, which may promotethe formation of a gel-like network on the leaf surface. Such a network may enhance the retention of water and nutrients, thereby increasing their persistence on the leaf surface. Consequently, the combined effects of moisture retention and nutrient supply may contribute to the observed increases in effective panicle number, filled grain number, seed-setting rate, and ultimately grain yield [36,37,38].

## 4. Conclusions

In this study, a foliar moisture-preserving synergist (FMPS) was developed using radiation-synthesized PAA-K as the matrix, with urea and glucose incorporated as nitrogen and carbon sources, respectively. Physicochemical characterization supported the successful incorporation of urea and glucose into the formulation and suggested the presence of intermolecular associations among the components. Under standard growth conditions, foliar application of FMPS increased grain yield by 16.5% compared with the control, which was accompanied by increases in effective panicle number (29.3%), filled grain number (18.4%), and seed-setting rate (4.0%). Based on physicochemical characterization and the detached-leaf water loss assay, we propose that the formulation may form a gel-like network on leaf surfaces upon drying, thereby enhancing moisture retention. However, this mechanism was not directly verified in the present study. Future studies should further investigate gel-network formation on leaf surfaces, nutrient delivery behavior, plant nutrient uptake, and the performance of FMPS under different environmental conditions. Overall, this study provides a feasible strategy for developing multifunctional foliar formulations to improve rice yield and establishes a basis for future investigations under field conditions and environmental variability.

## Data Availability

The original contributions presented in this study are included in the article. Further inquiries can be directed to the corresponding author.
